# Subxiphoid-subcostal thoracoscopic thymectomy for seropositive myasthenia offers equivalent remission rates and potentially faster recovery

**DOI:** 10.1093/icvts/ivab294

**Published:** 2021-11-18

**Authors:** Peng Cao, Shan Hu, Wensheng Qu, Kangle Kong, Peng Han, Jiaqi Yue, Yu Deng, Xiangning Fu, Fan Li, Bo Zhao

**Affiliations:** 1 Department of Thoracic Surgery, Tongji Hospital, Tongji Medical College, Huazhong University of Science and Technology, Wuhan, China; 2 Department of Neurology, Tongji Hospital, Tongji Medical College, Huazhong University of Science and Technology, Wuhan, China

**Keywords:** Myasthenia gravis, Thymectomy, Subxiphoid-subcostal, Thoracoscopic surgery, Prognostic factor

## Abstract

**OBJECTIVES:**

To compare the perioperative and follow-up outcomes of patients with myasthenia gravis (MG) receiving subxiphoid-subcostal or unilateral thoracoscopic thymectomy and to identify the factors affecting MG prognosis.

**METHODS:**

From January 2013 to December 2019, a total of 137 consecutive MG patients received subxiphoid-subcostal thoracoscopic thymectomy (STT, *n* = 65) or conventional unilateral thoracoscopic thymectomy (UTT, *n* = 72). The primary outcomes of this study were perioperative complications, duration and expenses of hospitalization, VAS score and complete stable remission (CSR).

**RESULTS:**

The patients receiving STT had significantly shorter drainage duration and postoperative hospital stay and lower hospitalization expenses (*P *<* *0.01). Pain scores on postoperative Days 1, 3, 7 and 14 were significantly lower in patients undergoing STT (*P *<* *0.01). The average follow-up was 54.3 ± 24.18 months, with a CSR rate of 30.6% and an overall effective rate of 87.3%. Through uni- and multivariable analyses, shorter symptom duration and Myasthenia Gravis Foundation of America (MGFA) class I were independent predictors for CSR in MG patients receiving thymectomy.

**CONCLUSIONS:**

The present study not only showed that STT was a safe and feasible technique for MG, with a potentially faster postoperative recovery, lower hospitalization expenses, less postoperative pain and equivalent remission rate, but also revealed that shorter symptom duration and MGFA class I were favourable prognostic factors for CSR.

**Figure:**
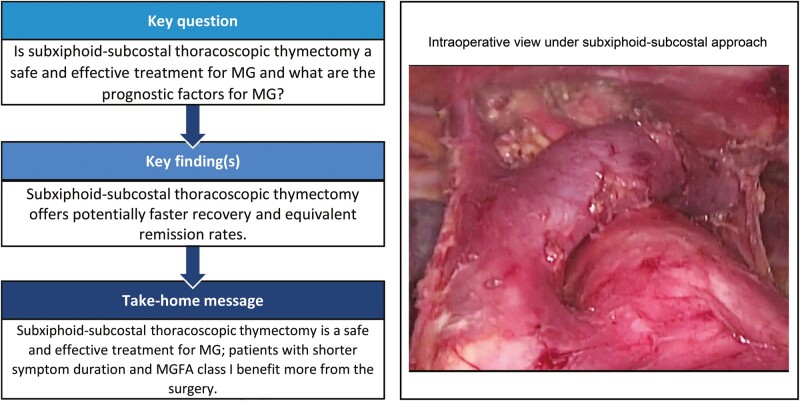

## INTRODUCTION

Myasthenia gravis (MG) is a long-lasting and rare autoimmune disease caused by neuromuscular junction dysfunction and characterized by weakness and abnormal muscle fatigue [1, 2]. The presence of thymic lesions in MG patients was previously described by Norris in 1936 [3]. According to a recent study, about 80% of MG patients had thymic lesions [4]. Since Blalock removed a thymic tumour in a 19-year-old MG patient and significantly relieved the symptoms in 1939 [5], thymectomy has been considered as an important component in the MG management. However, prognostic factors in MG patients undergoing thymectomy still remain uncertain.

Thymectomy with median sternotomy has been gradually replaced by minimally invasive surgery that has the benefits of less trauma, fewer complications and equivalent efficacy, including conventional video-assisted thoracoscopic surgery (VATS; unilateral or bilateral), robotic-assisted VATS and subxiphoid VATS [6–8]. The subxiphoid approach thymectomy was first reported by Kido in 1999 [9], and since then, subxiphoid thymectomy has been successfully applied in managing MG [8–10]. The subxiphoid approach is ideal and superior in terms of broader operative field, less intercostal neuralgia [10–12]. Hence, the aim of the present retrospective study was not only to evaluate perioperative outcomes of subxiphoid-subcostal thoracoscopic thymectomy (STT) versus unilateral thoracoscopic thymectomy (UTT) for MG but also to study the follow-up results and to identify the factors affecting MG prognosis.

## MATERIALS AND METHODS

### Ethical statement

This retrospective study was reviewed and approved by the Ethics Committee of Tongji hospital (Approval Number: TJ-C20190602). Written informed consent about operative techniques and data-use agreement was obtained from all patients.

### Patients

This was a retrospective study reviewing a prospectively collected database of MG patients undergoing STT or UTT from January 2013 to December 2019 at our department. All relevant data were within the Myasthenia Gravis Foundation of America. After being informed of the advantages and disadvantages of the two surgical methods, the final choice depended on the patient. The inclusion criteria were as follows: (i) Preoperative diagnosis of MG based on clinical symptoms, Tensilon test, electrophysiological test and serological test: detection of anti-acetylcholine-receptor antibodies; (ii) Diagnosis of thymic tumour by using chest computed tomography. And the exclusion criteria were: (i) Anterior mediastinal lesions larger than 6 cm that had invaded surrounding organs or neoadjuvant therapy had been given; (ii) Previous history of thoracic surgery, tuberculosis, preoperative pulmonary infection, diffuse emphysema; (iii) Coagulation dysfunction; (iv) Deformity of chest wall or spine; (v) American Society of Anesthesiologists (ASA) grade above III; (vi) Overweight (body mass index >30 kg/m^2^).

The clinical classification of MG was according to MGFA. The Quantitative Myasthenia Gravis (QMG) score was used to evaluate the severity of MG. Any patients with preoperative generalized symptoms, respiratory difficulties or bulbar symptoms were considered for pharmaceutical treatment, including pyridostigmine, prednisone or even plasmapheresis or intravenous immunoglobulins. All patients were discussed in a multidisciplinary board with neuroscientists with the aim to identify the surgical indication and to determine the appropriate timing of surgery based on the stability of MG symptoms. Thymectomy was merely performed when MG symptoms were significantly improved and daily dosage of prednisone was <20 mg/day, to reduce the risk of postoperative myasthenic crisis.

### Surgical procedures

**Figure 1:**
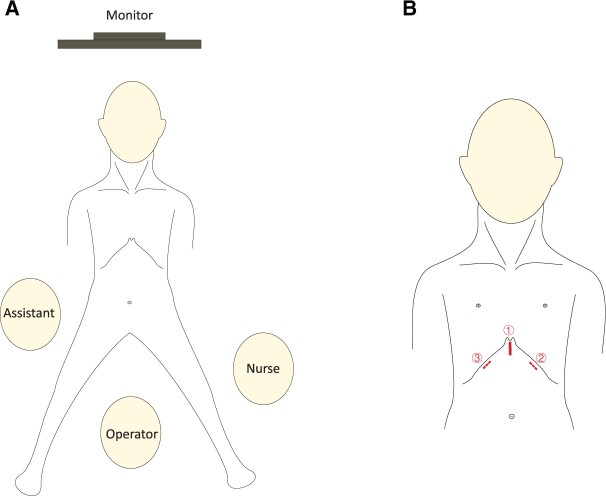
(**A**) Placement of the monitor, patient, operator, assistant and nurse in subxiphoid-subcostal thoracoscopic thymectomy; (**B**) Surgical incision in subxiphoid-subcostal thoracoscopic thymectomy: ① Subxiphoid incision for camera and CO_2_; ② Ultrasound scalpel; and ③ Grasping forceps.

**Figure 2:**
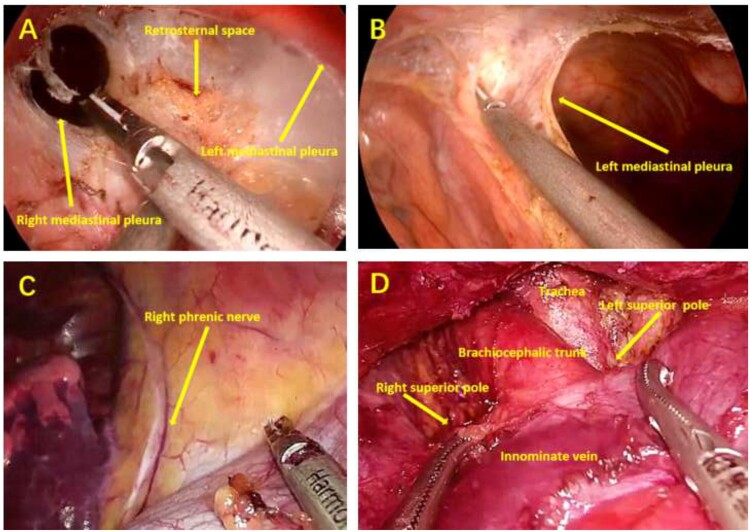
Intraoperative view under subxiphoid-subcostal thoracoscopic thymectomy. (**A**) Retrosternal space and bilateral mediastinal pleurae; (**B**) left mediastinal pleura; (**C**) right phrenic nerve; and (**D**) view of the cervical region.

Following the induction of general anaesthesia and mechanical ventilation with single-lumen endotracheal intubation, the patient was placed in a supine position with the legs spread, and a cushion was placed beneath the thoracic spine to elevate the chest (Fig. 1). The procedure of STT was similar to that reported previously [6]. During operation, the operator stood between the patients’ legs, while the assistant was on the right side of the patient to operate the camera (Fig. 1). A vertical skin incision of ∼3.0 cm was made first to expose the xiphoid and to serve as an observation port. The retrosternal space was created and enlarged by blunt separation with fingers. Under the guidance of the finger, two 0.5-cm extrapleural thoracic ports were created under the bilateral costal arches at the midclavicular lines to act as operating ports (Fig. 1). Thereafter, a 10-mm, 30-degree thoracoscope was introduced into the retrosternal space through the subxiphoid incision. Artificial pneumothorax was created with insufflation of carbon dioxide (CO_2_) under a positive pressure of 8-cm H_2_O which could shift the lung and diaphragm away to ensure a sufficient surgical field. The ultrasonic scalpel device was used for detachment, excision and coagulation. The bilateral mediastinal pleurae were incised first to expose the bilateral thoracic cavities. Then, the pericardial adipose tissue of bilateral diaphragmatic angle was dissected and bilateral phrenic nerves were identified. Along the anterior of bilateral phrenic nerves to the neck, the lateral edges of thymus and its surrounding adipose tissue were excised. The posterior edge of the thymus was separated from the pericardium to identify the innominate vein, and the junction of innominate vein and superior vena cava could be clearly exposed. After ligating the thymic veins, the thymus was stripped from the innominate vein. By grasping the thymus, the bilateral superior poles of thymus were easily dissected. The thymus in neck region was excised from the thyroid gland, innominate veins, brachiocephalic artery, aortopulmonary window and trachea (Fig. 2). The specimens were placed en-bloc in plastic bags and removed via the subxiphoid port. Haemostasis was assured and two 20-Fr drainage tubes were inserted into thoracic cavity via bilateral subcostal incisions.

**Figure 3:**
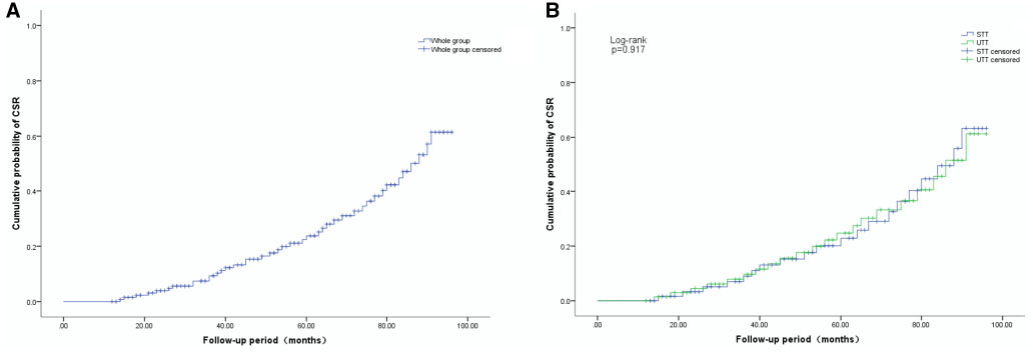
Cumulative probability of CSR after thymectomy. (**A**) Cumulative probability of CSR in the whole group; (**B**) Comparison of cumulative probability of CSR between STT and UTT. CSR: complete stable remission; STT: subxiphoid-subcostal thoracoscopic thymectomy; UTT: uilateral thoracoscopic thymectomy.

The procedure of UTT was similar to that reported previously [13]. Following the induction of general anaesthesia, a traditional double-lumen endotracheal intubation was used for UTT. The operation was performed with the patient in lateral decubitus position. The right-sided approach was used unless the tumour was predominantly located on the left. A 3-cm operation and camera ports were placed in the performed in the fifth and seventh intercostal space and at the anterior and mid-axillary line, respectively. Thymectomy was started from the phrenic nerve and diaphragm and proceeded gradually to the thoracic inlet. Then the innominate vein was identified and thymic veins were exposed and ligated using an ultrasonic scalpel. The dissection was continued up to the vicinity of contralateral phrenic nerve. The superior pole of thymus and fat tissues in neck region was dissected. Once the thymus and surrounding fat tissues were dissected free, it was placed into a plastic bag and removed through the incision. Two chest tubes were inserted and incisions were sutured.

### Postoperative management

Oxygen saturation and electrocardiography monitoring were performed in the early postoperative period. Chest radiography was performed at the first day postoperatively and after removal of drainage tube. Early extubation was encouraged after surgery, if the following extubation criteria were met: haemodynamic stability; stable spontaneous ventilation; arterial blood gas analysis results in normal range under low-flow oxygen inhalation; and clear consciousness. Early removal of drainage tube was also encouraged postoperatively, according to the following criteria: no observed air leak and total drainage <200 ml in 24 h; normal chest X-ray; normal vital signs. All patients were discharged without a chest tube *in situ*.

Under the guidance of the neurologists, the same medications used preoperatively were postoperatively restarted to control MG, but dosage and time of pyridostigmine and prednisone were adjusted according to postoperative clinical symptoms in critical patients. In general, no postoperative analgesics were used unless necessary. If pain was intolerable, low-dose diclofenac sodium could be used. Postoperative pain was monitored using the VAS score at 1, 3, 7, 14, 30, 90 and 180 days.

### Follow-up

All patients were regularly followed up, and the clinical data were obtained by reviewing the electronic medical records, nursing records, outpatient services or by direct telephone interview. The frequency of follow-up was once in the first month after surgery, once in the fourth month after surgery and then every 6 months in the following years. The drug management of MG patients was based on the patient’s symptoms and conducted under the guidance of experienced neurologists. Therapeutic responses were assessed according to the MGFA Post-Intervention Status [14]. The criteria defining the MGFA Post-Intervention Status required that the patient be examined by a skilled neurologist. An effective status in this study included complete stable remission (CSR), pharmacological remission (PR), minimal manifestation (MM), and improved (I), while an ineffective status included unchanged (U), worse (W), exacerbation (E) or death (D).

### Statistical analysis

All statistical analyses were performed using IBM SPSS Statistics for Windows, Version 22.0 (Armonk, NY: IBM Corp.). Continuous variables were presented as mean ± standard deviation, and a Student’s *t*-test was used to compare the means if the data were normally distributed; otherwise, the variables were displayed as median and range, and non-parametric Kruskal–Wallis test should be used. Categorical variables were presented as count and percentages, and were compared using chi-square test (or Fisher’s exact test) as appropriate. Survival curves were plotted for the patients using the Kaplan–Meier method and analysed using the log-rank test. Uni- and multivariable analyses were performed by Cox regression analysis to confirm the prognostic factors of CSR. Factors with a *P*-value of <0.2 were included for multivariable analysis to assess the independent prognostic factors affecting CSR. A *P*-value of <0.05 was considered statistically significant.

## RESULTS

### Baseline characteristics and perioperative results

A total of 137 patients between January 2013 and December 2019 were included in this analysis, of which 47.4% of patients received STT. Demographics for the two groups were shown in Table 1. There were no statistically significant differences between the two groups, including age, sex, body mass index, smoking, comorbidity, ASA score and MGFA clinical classification, maximal tumour size, World Health Organization (WHO) histologic type and pathological Masaoka–Koga stage of thymomas. However, there was a patient with type-C thymoma in STT group, but not in UTT group.

**Table 1:** Comparison of clinical and pathological data between two groups

Characteristics	STT（*n* = 65）	UTT (*n* = 72)	*P-*value
Age (years), median (range)	49 (18–67)	52 (18–71)	0.581
Sex, *n* (%)			0.667
Male	31 (47.7)	37 (51.4)	
Female	34 (52.3)	35 (48.6)	
BMI (kg/m^2^), median (range)	22.3 (16.1–29.8)	22.7 (15.9–30.0)	0.473
Smokers, *n* (%)	13 (22.3)	16 (21.6)	0.851
Comorbidity, *n* (%)			0.958
Extrathoracic malignancy	1 (1.5)	2 (2.8)	
Chronic obstructive pulmonary disease	9 (13.8)	11 (15.2)	
Hypertension	7 (10.8)	8 (11.1)	
Diabetes mellitus	3 (4.6)	5 (6.9)	
Cardiac diseases	4 (6.2)	4 (5.6)	
Others	1 (1.5)	2 (2.8)	
None	40 (61.6)	40 (55.6)	
ASA classification, median (range)	1 (1–3)	1 (1–3)	>0.999
QMG score	3.1 ± 2.11	3.4 ± 1.97	0.206
MGFA clinical classification, *n* (%)			0.799
I	29 (44.7)	35 (48.7)	
II	31 (47.7)	29 (40.2)	
III	4 (6.2)	6 (8.3)	
IV	1 (1.4)	2 (2.8)	
Maximal tumour size (cm)	3.27 ± 1.49	3.41 ± 1.38	0.592
Pathology, *n* (%)			0.946
Thymoma	19 (29.2)	22 (30.6)	
Thymic hyperplasia	27 (41.6)	32 (44.4)	
Thymic cyst	18 (27.7)	16 (22.2)	
Thymus atrophy	1 (1.5)	2 (2.8)	
WHO histologic type of thymomas, *n* (%)			0.960
A/AB	8 (12.3)	10 (13.9)	
B1/B2/B3	10 (15.4)	12 (16.7)	
C	1 (1.5)	0 (0.0)	
Masaoka–Koga stage, *n* (%)			0.291
I	15 (23.0)	18 (25.0)	
II	4 (6.2)	4 (5.6)	

ASA: American Society of Anesthesiologists; BMI: body mass index; MGFA: Myasthenia Gravis Foundation of America; QMG: Quantitative Myasthenia Gravis; WHO: World Health Organization.

All perioperative outcomes between STT and UTT groups were shown in Table 2. No statistically significant differences were found in operation time, intraoperative lowest pulse oxygen saturation, blood loss and perioperative complications between both groups. However, in UTT group, there was one patient with contralateral phrenic nerve paralysis postoperatively, but none in STT group. Two cases (2/72) in UTT group and 1 (1/65) in STT group were converted to thoracotomy due to chest adhesions or bleeding. The incidence of postoperative crisis and re-tracheal intubation was low in both groups. In addition, there was no 30-day mortality in both groups. But compared with the patients undergoing UTT, the patients receiving STT had significantly shorter drainage duration, postoperative hospital stay, and lower hospitalization expenses (*P *=* *0.006, *P *=* *0.002, *P *=* *0.004, respectively). VAS scores on postoperative Days 1, 3, 7 and 14 were significantly higher in patients undergoing UTT (*P *=* *0.002, *P *=* *0.003, *P *=* *0.005, *P *=* *0.007, respectively), but there was no significant difference on postoperative Days 30, 60 and 180 between the two groups (*P *=* *0.086, *P *=* *0.273, *P *=* *0.190, respectively).

**Table 2:** Perioperative outcomes between the STT and UTT groups

Variables	STT（*n* = 65）	UTT (*n* = 72)	*P-*value
Operation time, min	124.5 ± 40.82	131.8 ± 46.35	0.094
Intraoperative lowest SpO_2_, (%)	96.6 ± 6.37	95.2 ± 7.61	0.219
Blood loss (ml)	50.5 ± 32.71	61.3 ± 40.36	0.087
Intraoperative complications, *n* (%)			0.709
Conversion to thoracotomy	1 (1.5)	2 (2.8)	
Bleeding	1 (1.5)	1 (1.4)	
Drainage duration (days)	2.2 ± 1.13	3.5 ± 1.42	0.006^*^
Postoperative hospital stay (days)	3.4 ± 1.70	5.3 ± 2.94	0.002^*^
Hospitalization expenses (thousand USD)	5.2 ± 1.03	7.6 ± 1.91	0.004^*^
Postoperative complications, *n* (%)			0.987
Lung infection	2 (3.1)	3 (4.2)	
Chylothorax	1 (1.5)	1 (1.4)	
Pleural effusion or pneumothorax	1 (1.5)	2 (2.8)	
Phrenic nerve paralysis	0 (1.5)	1 (1.4)	
Wound infection	1 (1.5)	1 (1.4)	
Postoperative crisis	1 (1.5)	2 (2.8)	
Postoperative re-tracheal intubation	1 (1.5)	2 (2.8)	
30-day mortality	0 (0.0)	0 (0.0)	
Pain [0–10 (VAS-score)]			
POD 1	4.7 ± 0.52	6.4 ± 1.12	0.002^*^
POD 3	2.3 ± 0.39	4.6 ± 1.01	0.003^*^
POD 7	0.9 ± 0.31	2.1 ± 0.45	0.005^*^
POD 14	0.6 ± 0.27	1.1 ± 0.34	0.007^*^
POD 30	0.3 ± 0.19	0.5 ± 0.26	0.086
POD 60	0.2 ± 0.13	0.4 ± 0.21	0.273
POD 180	0.1 ± 0.09	0.2 ± 0.12	0.190

SpO_2_: pulse oxygen saturation; STT: subxiphoid-subcostal thoracoscopic thymectomy; USD: US dollars; UTT: uilateral thoracoscopic thymectomy.

### Neurological outcome

At the time of completion of data gathering for analysis, 3 (4.2%) patients were lost to follow-up evaluation, including 1 in STT group and 2 in UTT group. The remaining 134 patients had a median follow-up period of 52 months (range: 12–96 months; mean: 54.3 ± 24.18 months). Of the 134 patients, a total of 30.6% patients achieved CSR, 23.6% patients achieved PR, 22.4% patients had MM, 14.4% patients improved, 6.7% patients were unchanged, 3.0% patients were worse, in 2 patients (1.5%) there was exacerbation and 2 patients (1.5%) died of respiratory failure caused by myasthenia crisis during the follow-up period (1 in STT group and 1 in UTT group) (Table 3). Overall, treatment was effective in 87.3% (117/134) patients (CSR + PR + MM + I) and ineffective in 12.7% (17/134) patients (U + W + E + D). There were no significant differences in CSR, effective status and cumulative probability of CSR between STT and UTT (*P *=* *0.985, *P *=* *0.917, respectively) (Table 3 and Fig. 3B).

**Table 3:** The MGFA-PIS of patients after thymectomy

MGFA-PIS	Overall (*n* = 134)	STT （*n* = 64）	UTT (*n* = 70)	*P-*value
Effective, *n* (%)	117 (87.3)	55 (85.9)	62 (88.6)	0.985
CSR	41 (30.6)	20 (31.2)	21 (30.0)	
PR	32 (23.9)	15 (23.4)	17 (24.3)	
MM	30 (22.4)	14 (21.9)	16 (22.9)	
I	14 (10.4)	6 (9.4)	8 (11.4)	
Ineffective, *n* (%)	17 (12.7)	9 (14.1)	8 (11.4)	0.997
U	9 (6.7)	5 (7.8)	4 (5.7)	
W	4 (3.0	2 (3.1)	2 (2.9)	
E	2 (1.5)	1 (1.6)	1 (1.4)	
D	2 (1.5)	1 (1.6)	1 (1.4)	

Effective: CSR + PR + MM + I; Ineffective: U + W + E + D.

CSR: complete stable remission; D: death; E: exacerbation; I: improved; MM: minimal manifestation; PIS: post-intervention status; PR: pharmacological remission; STT: subxiphoid-subcostal thoracoscopic thymectomy; U: unchanged; UTT: uilateral thoracoscopic thymectomy; W: worse.

Possible prognostic factors of CSR for MG patients after thymectomy were evaluated (Tables 4 and 5). Univariable analysis (*n* = 134) showed that onset age [<40 years vs ≥40 years, hazard ratio (HR) = 2.073, *P *=* *0.048], symptom duration (<1 year vs ≥1 year, HR = 4.106, *P *=* *0.005) and MGFA clinical classification (I versus II and above, HR = 3.154, *P *=* *0.029) were predictors of response to thymectomy in MG patients, whereas sex, pathology and surgical approach were not significantly correlated with neurological outcome. In the multivariable analysis, shorter symptom duration (<1 year: HR = 3.908, *P *=* *0.019) and MGFA class I (HR = 3.271, *P *=* *0.035) were considered as independent favourable predictors for a higher CSR in MG patients receiving thymectomy, while the onset age was not considered as a significant prognostic factor (<40 years: HR = 1.973, *P *=* *0.061).

**Table 4:** Univariable analysis for predictors of CSR after thymectomy

Variables	Total (*n* = 134)	CSR (*n* = 41)	HR (95% CI)	*P*-value
Sex				
Male	65	20 (30.8%)	1.075 (0.581–1.673)	0.839
Female	69	21 (30.4%)	1	
Onset age				
<40 years	75	26 (34.7%)	2.073 (1.084–3.965)	0.048^*^
≥40 years	59	15 (25.4%)	1	
Symptom duration				
<1 year	81	30 (37.0%)	4.106 (1.628–9.750)	0.005^*^
≥1 year	53	11 (20.2%)	1	
MGFA clinical classification				
I	63	24 (38.1%)	3.154 (1.260–6.729)	0.029^*^
II and above	71	17 (24.0%)	1	
Pathology				
Non-thymoma	93	30 (32.3%)	1.472 (0.906–3.281)	0.382
Thymoma	41	11 (26.7%)	1	
Surgical approach				
STT	64	20 (31.3%)	1.193 (0.703–2.065)	0.947
UTT	70	21 (30.0%)	1	

CI: confidence interval; CSR: complete stable remission; HR: hazard ratio; MGFA: Myasthenia Gravis Foundation of America; STT: subxiphoid-subcostal thoracoscopic thymectomy; UTT: uilateral thoracoscopic thymectomy.

**Table 5:** Multivariable analysis for predictors of CSR after thymectomy

Variables	SE	Wald	HR	95% CI	*P*-value
Onset age					
<40 years vs ≥40 years	0.595	3.663	1.973	0.927–3.892	0.061
Symptom duration					
<1 year vs ≥1 year	0.651	12.719	3.908	1.583–9.6813	0.019^*^
MGFA clinical classification					
I versus II and above	0.637	11.206	3.271	1.295–6.840	0.035^*^

CI: confidence interval; CSR: complete stable remission; HR: hazard ratio; SE: standard error.

## DISCUSSION

In this study, we evaluated the perioperative and follow-up results of patients receiving STT and UTT and found that STT was a safe and feasible technique for MG, with a faster recovery, lower hospitalization expenses, less postoperative pain and equivalent remission rate. The results also suggested that MG patients with shorter symptom duration and MGFA class I benefitted more from the surgery.

Over the past several decades, multiple surgical techniques have been described for thymectomy in treating MG and thymic tumours, including trans-sternal thymectomy and now popular minimally invasive surgery, as long as all thymus and surrounding fat were removed [2, 14]. With advances of surgical instruments and techniques, VATS thymectomy has become a popular alternative to trans-sternal thymectomy in recent years [15]. At present, the lateral approach is the main choice in various approaches of VATS thymectomy. However, the lateral approach has its limitations, including difficulty in identifying the contralateral phrenic nerve, narrow operative field in neck region, inadequate removal of fat on the contralateral side and intercostal nerve paralysis or neuralgia [16]. Although the bilateral approach could provide a wide surgical field and expose bilateral phrenic nerves, it leads to more incisions and traumas. Although there were efforts to reach a consensus regarding the standard VATS thymectomy, no consensus has been available so far [17], and the choice of surgical approach is usually based on the experience and preference of the surgeon.

Hsu [6] and Suda *et al.* [12, 16] previously reported that subxiphoid VATS thymectomy without intercostal incisions had several advantages. First, the excellent operative field offered by a subxiphoid approach could easily help to confirm the location of the superior poles of thymus and bilateral phrenic nerves. The broad visualization allowed the maximum resection of thymus and its surrounding adipose tissues and reduced the chance of accidental vessel laceration or phrenic nerve injury. Secondly, double-lumen intubation and single-lung ventilation were necessary for UTT, which met the needs of intraoperative lung ventilation and adequate surgical field [7, 8, 10–12]. However, as shown in our study, STT could be successfully performed under a single-lumen intubation. Although there was no statistical difference in the lowest intraoperative pulse oxygen saturation between the two groups in our study [96.6 ± 6.37 vs 95.2 ± 7.61, (%)], we believed that for patients who cannot tolerate single-lung ventilation because of impaired lung function or double-lumen endotracheal intubation, it was safe and feasible to use a single-lumen intubation for STT. Third, previous studies had shown that the postoperative pain of STT was significantly lower than that of lateral approach by avoiding intercostal incision and nerve damages [8, 10, 11, 16]. Our present research also revealed that pain scores on postoperative Days 1, 3, 7 and 14 were significantly lower in STT group (*P *<* *0.01). By comparing the postoperative VAS scores, the pain scores of our two-port technique were similar to that of three-port VATS thymectomy [10]. Further, compared with UTT, STT had shorter drainage duration and postoperative hospital stay and lower hospitalization expenses [10, 11, 16].

However, the STT also has disadvantages. Like all thoracoscopic surgery, even with the presence of artificial pneumothorax, an excessively high body mass index would lead to a narrow surgical field and further make surgery difficult, as well as creating a problem of mutual interference between surgical instruments. In addition, for patients with thymic tumours invading surrounding tissues, organs or large vessels, STT may be unsuitable, and open surgery should be recommended. In other words, STT needs to be used selectively.

Therapeutic thymectomy has been a mainstay in the treatment of MG [1, 2, 18]. In 2016, Wolfe *et al.* [18] conducted a multicentre and randomized trial of thymectomy in MG, which provided evidence that thymectomy improved clinical outcomes in MG patients. In the present analysis of 137 MG patients, we noted that MG patients undergoing thymectomy could achieve a CSR rate of 30.6%, which was comparable to that reported in the literature [19–24]. Previous studies had found that there was no significant difference in CSR between thoracoscopic and trans-sternal thymectomy [22, 24]. There was a study showing that the CSR of subxiphoid thymectomy was comparable with VATS thymectomy, our results also confirmed this finding [19]. This result showed that the effect of subxiphoid thymectomy for MG was worthy of recognition. However, there are still few studies on the effect of subxiphoid thymectomy on CSR of MG, so this result still needs to be verified by a large sample of randomized controlled trials.

Identifying prognostic factors of MG plays an important role in treatment decisions. Many factors influencing the CSR had been reported, including the sex, onset age, symptoms duration, MGFA clinical classification, coincidence of thymoma and so on. However, data were not only limited but also contradictory. It was reported that male patients were more likely to achieve a favourable clinical outcome than female [22], but other studies revealed that sex was not related to clinical outcomes [21, 23]. Similarly, our results showed that sex had no significant adverse effect on clinical outcome. Some researchers reported that MG patients with early-onset age had a better clinical outcome [19, 24, 25]. Though the present study also showed a higher CSR with an early onset age, but there was no statistical significance in multivariable analysis. Both uni- and multivariable analyses of the current study confirmed previous findings, indicating shorter symptoms duration as a predictor of satisfactory response to thymectomy [26, 27]. The presence of thymoma was generally considered an adverse prognostic factor for MG [19, 22]. However, there were also some reports showing that thymectomy could achieve the same effect, whether patients combined with or without thymoma [28]. In our follow-up study, the prognosis of MG patients with thymoma was equivalent to that of patients without thymoma. As reported in the previous literature, our results also showed that preoperative MGFA class I was an independent favourable predictor of CSR [19, 23].

The single-centre retrospective nature of the study represents its major obvious limitation. All patients were selected from only one clinical centre, and thus, intrinsic bias may exist, including the surgeons’ preferences. In addition, we did not perform propensity score matching analysis because of the small sample size. Therefore, further studies with larger samples multicentre prospective randomized controlled trials should be conducted.

## CONCLUSION

In conclusion, the present study showed that STT is a safe and effective technique for the management of MG patients, with a shorter drainage duration and postoperative hospital stay, lower hospitalization expenses, less postoperative pain and equivalent CSR rate. Although our research was limited by being a retrospective study, it still revealed that shorter symptom duration and MGFA class I were favourable prognostic factors for CSR.

**Conflict of** **interest:** none declared. 

### Author contributions

**Peng Cao:** Data curation; Methodology; Software; Writing—original draft. **Shan Hu:** Data curation. **Wensheng Qu:** Conceptualization; Data curation. **Kangle Kong:** Formal analysis. **Peng Han:** Data curation. **Jiaqi Yue:** Software. **Yu Deng:** Conceptualization. **Xiangning Fu:** Project administration. **Fan Li:** Conceptualization; Validation; Writing—review & editing. **Bo Zhao:** Conceptualization; Resources; Supervision, Writing—review & editing.

### Reviewer information

Interactive CardioVascular and Thoracic Surgery thanks Tibor Krajc, William Grossi, Rui Haddad and the other anonymous reviewers for their contribution to the peer review process of this article.

## ABBREVIATIONS

CSR: Complete stable remission
D: Death
E: Exacerbation
HR: Hazard ratio
I: Improved
MG: Myasthenia gravis
MGFA: Myasthenia Gravis Foundation of America
MM: Minimal manifestation
PR: Pharmacological remission
STT: Subxiphoid-subcostal thoracoscopic thymectomy
U: Unchanged
UTT: Unilateral thoracoscopic thymectomy
VATS: Video-assisted thoracoscopic surgery
W: Worse
